# Fetal Fascial Reinforcement Development: From “a White Tablet” to a Sculpted Precise Organization by Movement

**DOI:** 10.3390/biology11050735

**Published:** 2022-05-11

**Authors:** Carmelo Pirri, Lucia Petrelli, Albert Pérez-Bellmunt, Sara Ortiz-Miguel, Caterina Fede, Raffaele De Caro, Maribel Miguel-Pérez, Carla Stecco

**Affiliations:** 1 Department of Neurosciences, Institute of Human Anatomy, University of Padova, 35121 Padova, Italy; lucia.petrelli@unipd.it (L.P.); caterina.fede@unipd.it (C.F.); rdecaro@unipd.it (R.D.C.); 2 Basic Sciences Department, Universitat Internacional de Catalunya, 08017 Barcelona, Spain; aperez@uic.cat (A.P.-B.); saraormi94@gmail.com (S.O.-M.); mabelmig@gmail.com (M.M.-P.); 3 ACTIUM Functional Anatomy Group, Faculty of Medicine and Health Sciences, 08195 Barcelona, Spain; 4 Unit of Human Anatomy and Embryology, Department of Pathology and Experimental Therapeutics, Faculty of Medicine and Health Sciences (Bellvitge Campus), University of Barcelona, 08907 Hospitalet del Llobregat, Spain

**Keywords:** fetus, fascia development, retinacula, gross functional movements

## Abstract

**Simple Summary:**

Nowadays, the number of studies concerning fasciae is increasing, but few studies focus on fetal fasciae development and there is no study on the retinacula. The latter are fascial reinforcements with a crucial role in proprioception and coordination. We aimed to identify their structural organization by qualitative and quantitative assessments, to establish their role in myofascial development, highlighting their appearance and organization. Our data strongly suggest that the movement models the fascial reinforcements, structuring the fascial system, particularly at the end of the pregnancy.

**Abstract:**

Fasciae have received much attention in recent years due to their important role in proprioception and muscular force transmission, but few studies have focused on fetal fasciae development and there is no study on the retinacula. The latter are fascial reinforcements that play a key role in proprioception and motor coordination. Furthermore, it is still unclear if they are genetically determined or if they are defined by movements, and if they are present during gestation or if they appear only later in the childhood. We aim to identify their structural organization by qualitative and quantitative assessments to establish their role the myofascial development, highlighting their appearance and organization. Samples from the wrist retinacula, posterior forearm, ankle retinacula, anterior leg, iliotibial tract and anterior thigh of six fetus body donors (from 24th to 40th week of gestation) and histological sections were obtained and a gross anatomy dissection was performed. Sections were stained with hematoxylin-eosin to observe their overall structure and measure their thicknesses. Using Weigert Van Gieson, Alcian blue and immunostaining to detect Hyaluronic Acid Binding Protein (HABP), Collagens I and III (Col I and III) were realized to assess the presence of elastic fibers and hyaluronan. This study confirms that the deep fasciae initially do not have organized layers and it is not possible to highlight any reinforcement. The fascial development is different according to the various area: while the deep fascia and the iliotibial tract is already evident by the 27th week, the retinacula begin to be defined only at the end of pregnancy, and their complete maturation will probably be reached only after birth. These findings suggest that the movement models the retinacula, structuring the fascial system, in particular at the end of pregnancy and in the first months of life. The fasciae can be imagined, initially, as “white tablets” composed of few elastic fibers, abundant collagens and HA, on which various forces, u movements, loads and gravity, “write their history”.

## 1. Introduction

Nowadays, there is a lot of evidence on the role of fasciae in anatomical, histological, clinical and rehabilitative fields [1,2,3]. Fasciae make up a dynamic structure that is reinforced by some fibrous bundles, such as the iliotibial tract and the retinacula. The latter are present around all the joints, but the most studied retinacula are the ankle and wrist retinacula. The retinacula are classically [4] considered as a pulley that maintain the tendons adherent to the underlying bone during all the movements of the near joint. Additionally, they are considered to be important elements for joint stability, connecting different bony structures [5,6,7,8]. The fibrosis of the retinacula could also lead to increased friction of the tendons as they slide in and out of their groove, eventually resulting in damage and the inflammation of the tendons [5,6]. However, in 1984, Viladot et al. affirmed that “the retinacula are thin and easily extensible, so they have a modest effect on the mechanical stability of the ankle, while they could have an important role in the proprioception. Besides, the peroneal retinacula are stretched by the inversion of the ankle joint, activating the reflex contraction of the peroneal muscles” [9]. From a histological point of view, few descriptions can be found in the literature. Klein et al. [10] stated that “the wrist and the ankle retinacula were formed by three histological layers (i.e., inner gliding layer, thick middle layer containing collagen bundles and outer layer of loose connective tissue containing vascular channels)”. They also mentioned that “elastic fibers were few and scattered among the fibroblasts”. These findings were also confirmed by Stecco et al. [11], who demonstrated that “the extensor ankle retinacula are formed of 2–3 layers of parallel collagen fiber bundles, densely packed with a little loose connective tissue, without elastic fibers but with many nerve fibers and corpuscles” [9,11]. Additionally, the deep fascia also presents some longitudinal reinforcements, such as the iliotibial band. 

Despite the fact that fascial reinforcement seems to play a key role in motor coordination and proprioception, there is no clear evidence about their ontogeny. To date, there have been few studies on the embryogenesis of the fascial layers, but no studies have focused on the retinacula [12,13,14,15]. Blasi et al. described, in a histological study, in a qualitative and morphometric manner the thickness of the connective tissue between subcutaneous adipose tissue and the underlying muscle (without a differentiation between superficial and deep fasciae) in the gestation period between the 22 and 39 weeks, showing the presence of connective tissue that is topographically and morphologically equivalent to adult deep fasciae [12]. Moreover, the authors highlighted the presence of Tcf4 + fibroblasts in the deep fascia, suggesting its crucial role in muscle morphogenesis [12]. Abe et al. realized the histological examination of thoracolumbar fascia in 25 embryos and fetus at 6–37 weeks, highlighting, within the prenatal life, the drastic change which occurs in inter-fascial connections and their topographical relation to muscle [13]. Cho et al. examined, in a descriptive manner, non-decalcified histological sections of the arms and thighs of 20 human fetuses aged 25–33 weeks [14], highlighting that the advanced lamination of deep fasciae did not usually accompany an increase in the thickness of the subcutaneous tissue: both seemed to be independent [14]. 

The current study set out to assess and characterize the deep fasciae and retinacula in different regions and at different stages of human fetal development. We aim to identify and measure the thickness of these structures, realizing a description in qualitative and quantitative manners of fascial fetal development.

## 2. Materials and Methods

Six fetal cadaveric body donors (24–40 weeks of gestation; 4 females and 2 males) were evaluated in this study. Fetuses were cryopreserved at the temperature of −20 °C, for a period ranging from a few weeks to six months, not embalmed. Specimens were processed by dissection on one side, and on the contralateral side samples for the microscopic evaluation were obtained. The macroscopic study and the samples were collected at the Universitat Internacional de Catalunya, the histological study at the University of Padova. Full thickness samples from the skin to the bone were obtained from the wrist, the forearm, the ankle, the leg, the lateral and the anterior regions of the thigh (from a topographically homologous site to the adult deep fasciae and retinacula). 

### 2.1. Gross Anatomy 

**Figure 1:**
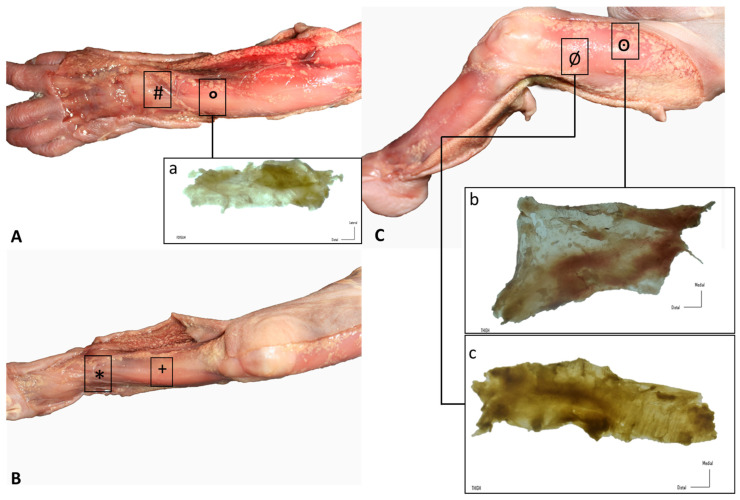
Gross anatomy view of the different samples (rectangles): (**A**) upper limb: (#) extensor retinaculum of the wrist and (**a**, °) dorsal antebrachial fascia; (**B**) lower limb, leg: (+) anterior crural fascia and (*) extensor retinaculum of the ankle; (**C**) lower limb, thigh: (**b**, ꙩ) anterior and (**c**, Ø) lateral deep fasciae.

To study the disposition of the different anatomical layers, dissections were made using the classical anatomical approach. First, a longitudinal incision was made, followed by two transverse incisions at the cranial and distal aspects of the longitudinal incision. The regions of study were the retinacula of the wrist and of the ankle, the anterior thigh fascia and the iliotibial tract, the leg and the forearm. Then, the skin was carefully dissected apart from the subcutaneous adipose tissue and superficial fascia, which was also carefully removed to reveal the deep fasciae and retinacula. A qualitative analysis of fascial continuity was performed (Figure 1).

### 2.2. Histological Study 

Full-thickness samples were obtained and dissected by the en bloc removal of the regions of study. After removal, the samples were mounted on cardboard to avoid deformation artifacts, fixed in 10% formalin solution and sent to the University of Padua for histological study. A small piece of paper indicating sample orientation was placed on the sample to guide the histological slice. At the University of Padua, the samples were re-fixed in 10% formalin solution and embedded in paraffin, with attention being paid to obtain full-thickness sections from the skin to the deep layer. Sections that were 5 μm thick were stained with hematoxilyn-eosin, Weigert Van Gieson and Alcian blue, and three sections per specimen were considered for the morphometric and morphological analyses [16]. All preparations were observed under a DM4500-B light microscope (Leica Microsystems, Wetzlar, Germany) and the image of six field, at a magnification of 1.6×, were recorded in full color (24 bits) by a digital camera (DFC 480, Leica Microsystems, Wetzlar, Germany). 

Computer-assisted image analysis was then used to measure the fascial thicknesses and the ImageJ software [17] (freely available at http://rsb.info.nih.gov/ij/ (accessed on 13 May 2021)) was used to perform this analysis. To eliminate the influence of thickness variations, three equidistant regions of interest per image for fascial reinforcements were measured; in each of them, three points representing the best visibility for each fascial layer were measured. Morphometric measurements of the deep fasciae and retinacula of the full-thickness specimens were recorded. The mean values and standard deviations of the thickness measurements were calculated. 

### 2.3. Immunocytochemistry to Detect Hyaluronic Acid-Binding Protein (HABP)

Endogen peroxidases were blocked with 0.5% H_2_O_2_ in PBS for 10 min at room temperature. After repeated washing in distilled water, the samples were then pre-incubated with a blocking buffer (0.2% bovine serum albumin, BSA, and 0.2% Triton-X, in PBS) for 60 min at room temperature. The samples were then incubated in hyaluronic acid-binding protein (HABP), Bovine Nasal Cartilage, Biotinylated, (Merck Life Science S.r.l., Milano, Italy), diluted 1:1000 in the same pre-incubation buffer, and maintained overnight at 4 °C. After repeated PBS washing, samples were incubated for 30 min in peroxidase-conjugated streptavidin (Jackson ImmunoResearch Laboratories, Inc., West Grove, PA, USA), diluted 1:250 in the same pre-incubation buffer. The reaction was then developed with 3,3′-diaminobenzidine (Liquid DAB plus substrate Chromogen System kit; Dako, Glostrup, Denmark) and stopped with distilled water. Negative controls were carried out by omitting incubation with HABP, confirming the specificity of the immunocytochemistry analysis with HABP.

### 2.4. Immunocytochemistry to Detect Collagen Types I and III

After blocking of endogen peroxidase by 0.5% H_2_O_2_ in PBS for 10 min at room temperature and repeated washings in PBS, samples were pre-incubated with a blocking 179 buffer (0.1% BSA in PBS) for 60 min at room temperature, and then incubated in goat anti-collagen-Type I, (1:400, SouthernBiotech, Birmingham, AL, USA), or rabbit anti-collagen-III, N-terminal antibody (1:100, Abcam, Cambridge, UK), overnight at 4 °C. After repeated PBS washings, samples were maintained for 1 h in secondary antibody (Collagen Type I), peroxidase Rabbit Anti-Goat and (Collagen III) peroxidase-Goat Anti-Rabbit (1:300, Jackson Immu-noResearch Laboratories, Inc., West Grove, PA, USA), and then washed in PBS. The reaction was developed with 3,3′-diaminobenzidine (Liquid DAB plus substrate Chromogen System kit; Dako, Glostrup, Denmark). Negative controls were conducted by the omission of the primary antibody, confirming the specificity of the immunostaining. 

## 3. Results

### 3.1. Gross Anatomy 

Similar findings were obtained during the dissection of all the specimens. The skin was very easily dissected from the subcutaneous adipose tissue, which constituted a very thin layer. Adipose tissue was mostly located around nerves and showed a granular pattern that tended to progressively increase. Nerves were easily observed and easy to dissect. A continuous deep fascia-like tissue was found underlying the subcutaneous adipose tissue; it was continuous in all regions of study, packing all the muscles. In the wrist and the ankle, we clearly recognized deep fascia-like connective tissues but they were not clear fascial reinforcements. The deep fascia tissues were very easily separated from the underlying muscles in all regions of study (wrist, forearm, thigh, leg and ankle) (Figure 1).

### 3.2. Histological Study 

**Figure 2:**
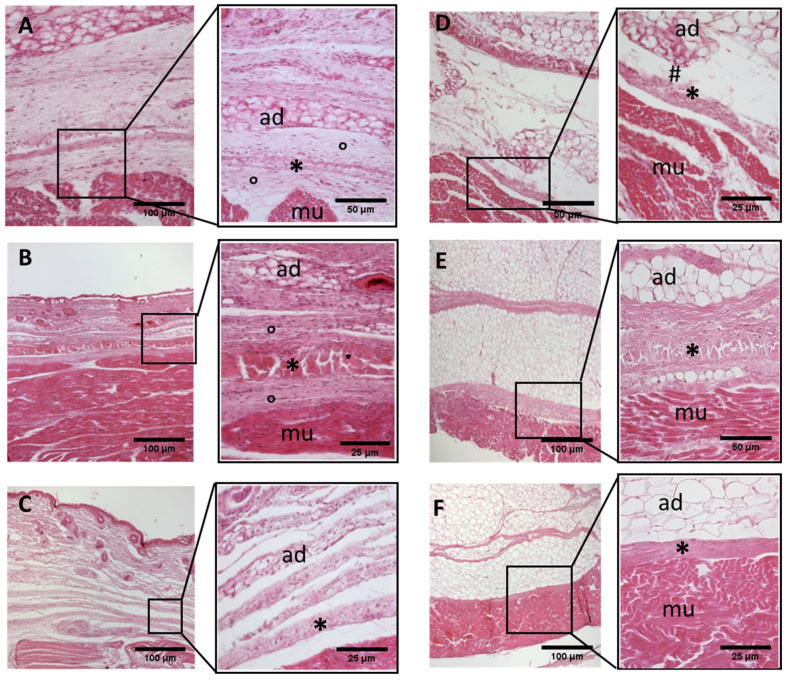
**Histological images (hematoxilyn-eosin) of lower limb, thigh: anterior fascia development.** (**A**): 24 weeks; (**B**): 27 weeks; (**C**): 29 weeks; (**D**): 36 weeks; (**E**): 38 weeks; (**F**): 40 weeks. (*): deep fascia; (°): mesenchymal cells; (#): areolar tissue; (mu): muscle; (ad): adipose tissue. The first part of gestation is distinguished (**A**–**C**) by an irregular connective tissue with a huge cellularity and the absence of sublayers, whilst the second part of the gestation (**D**–**F**) showed up with a denser connective tissue, which was better organized in the fascial layers.

**Figure 3:**
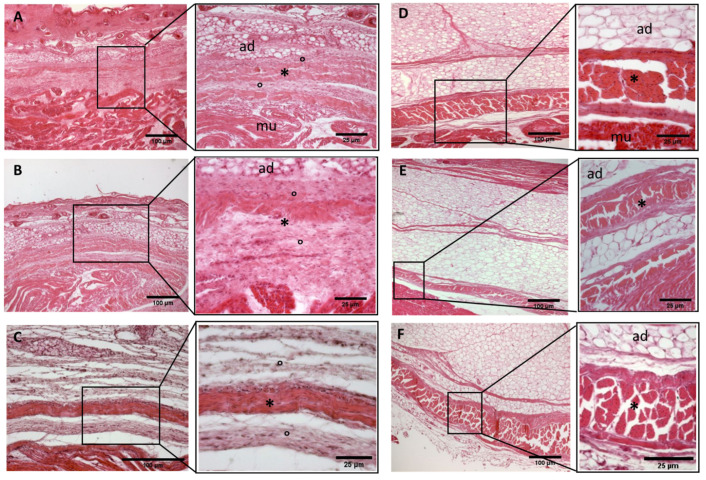
**Histological images (hematoxilyn-eosin) of lower limb, thigh: lateral fascia (iliotibial tract) development.** (**A**): 24 weeks; (**B**): 27 weeks; (**C**): 29 weeks; (**D**): 36 weeks; (**E**): 38 weeks; (**F**): 40 weeks. (*): deep fascia; (°): mesenchymal cells; (mu): muscle; (ad): adipose tissue. The first part of gestation is distinguished (**A**–**C**) by an irregular connective tissue with a huge cellularity without clear organization in layers, whilst the second part of the gestation (**D**–**F**) showed up with a denser connective tissue, which was better organized in the fascial layers. The middle layer was the most prominent and consisted of compact bundles of collagen fibers, while the outermost layers show a woven-like and thin pattern (**F**).

**Figure 4:**
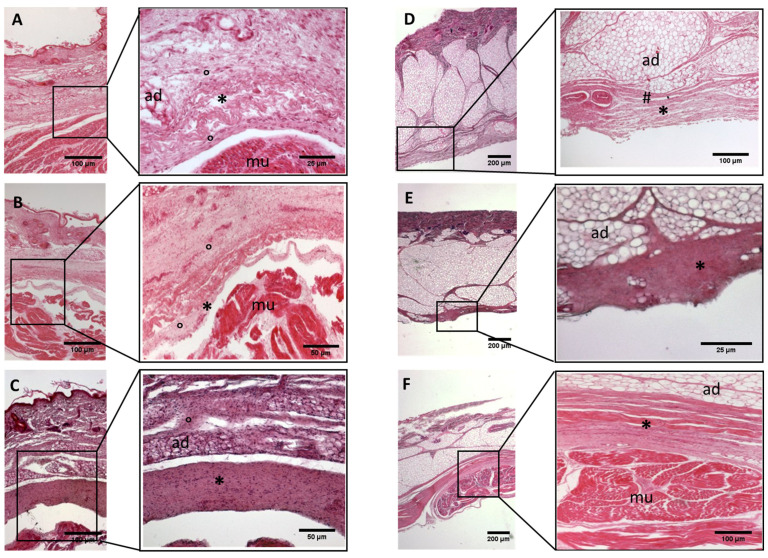
**Histological images (hematoxilyn-eosin) of lower limb, leg: crural fascia development**. (**A**): 24 weeks; (**B**): 27 weeks; (**C**): 29 weeks; (**D**): 36 weeks; (**E**): 38 weeks; (**F**): 40 weeks. (*): deep fascia; (°): mesenchymal cells; (#): areolar tissue; (mu): muscle; (ad): adipose tissue. The first part of gestation is distinguished (**A**–**C**) by an irregular connective tissue without clear organization in layers, whilst the second part of the gestation (**D**–**F**) showed up with a denser connective tissue, and was better organized in the fascial layers.

**Figure 5:**
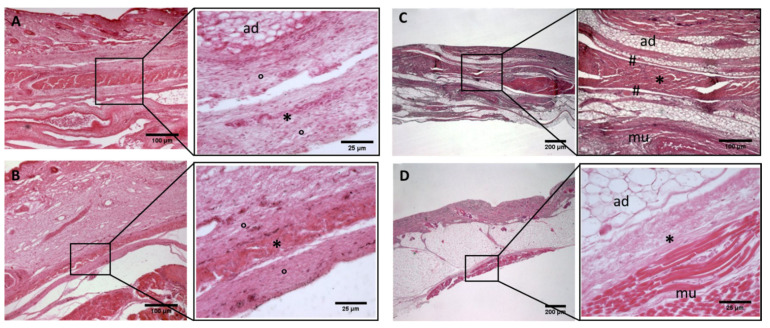
**Histological images (hematoxilyn-eosin) of lower limb: extensor retinaculum development**. (**A**): 24 weeks; (**B**): 29 weeks; (**C**): 38 weeks; (**D**): 40 weeks. (*): deep fascia; (°): mesenchymal cells; (#): areolar tissue; (mu): muscle; (ad): adipose tissue. The first part of gestation is distinguished (**A**,**B**) by an irregular connective tissue, whilst the second part of the gestation (**C**,**D**) showed up with a denser connective tissue, which was better organized in the fascial layers.

**Figure 6:**
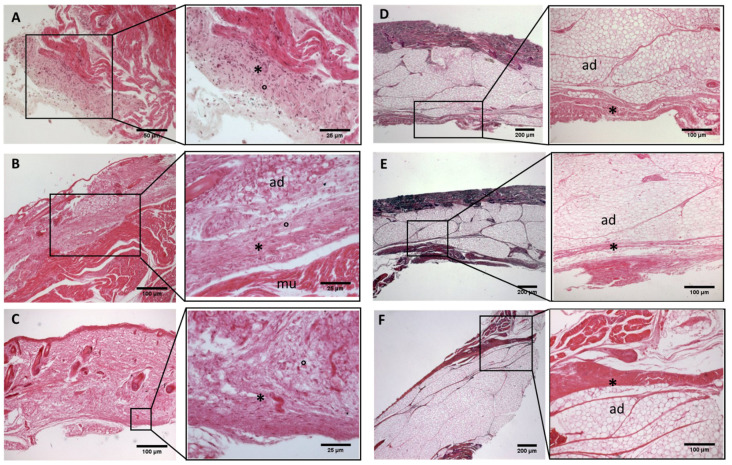
**Histological images (hematoxilyn-eosin)of upper limb: antebrachial fascia development**. (**A**): 24 weeks; (**B**): 27 weeks; (**C**): 29 weeks; (**D**): 36 weeks; (**E**): 38 weeks; (**F**): 40 weeks. (*): deep fascia; (°): mesenchymal cells; (mu): muscle; (ad): adipose tissue. The first part of gestation is distinguished (**A**–**C**), by an irregular connective tissue with a huge cellularity without clear organization in layers, whilst the second part of the gestation (**D**–**F**) showed up with a denser connective tissue, which was better organized in the fascial layers.

**Figure 7:**
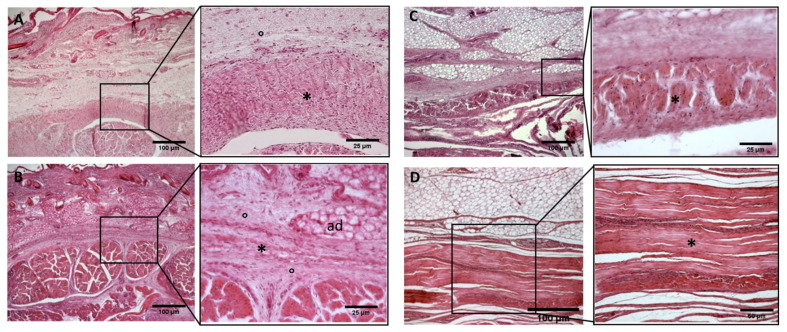
**Histological images (hematoxilyn-eosin) of upper limb: extensor retinaculum development**. (**A**): 24 weeks; (**B**): 27 weeks; (**C**): 38 weeks; (**D**): 40 weeks. (*): deep fascia; (°): mesenchymal cells; (mu): muscle; (ad): adipose tissue. The first part of gestation is distinguished (**A**,**B**) by an irregular connective tissue, whilst the second part of the gestation (**C**,**D**) showed up with a denser connective tissue, which was better organized in the fascial layers.

**Figure 8:**
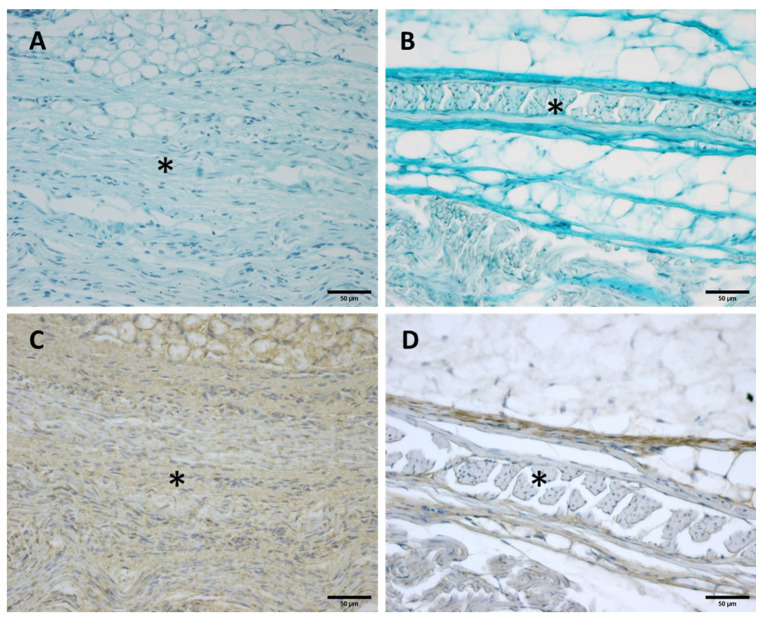
Alcian Blue staining and Immunostaining to detect hyaluronic acid-binding protein (HABP): anterior part of the deep fascia of thigh: (**A**,**C**): 24 weeks; (**B**,**D**): 38 weeks. (*): deep fascia. The first part of the gestation is characterized, starting from the 24th week, by less dense and irregular connective tissue with HA distributed in a disorganized manner, whilst the second part of the gestation, from the 36th week, presented with a denser and compact connective tissue with HA distributed in an organized structure which tends to diminish in quantity.

A continuous sheet of connective tissue was observed between the subcutaneous tissue and the underlying muscle tissue across all samples and in all the regions of study, with some differences (Figure 2, Figure 3, Figure 4, Figure 5, Figure 6, Figure 7 and Figure 8). The morphometric thickness parameters of the connective tissue structures are shown for all samples in Table 1.

− Week 24: In all samples, a singular layer of mesenchymal irregular connective tissue was observed. The deep fascia was clearly identified (Figure 2A, Figure 3A, Figure 4A, Figure 5A and Figure 6A) but it was not organized in layers and the fascial thickness is huge in all samples, 531.6 ± 178.4 µm, except for the extensor retinaculum of the upper limb 112.40 ± 6.1 µm (Figure 7A). In the deep fascia, a huge cellularity without clear organization was evident. Hematoxylin-eosin demonstrated the presence of a high number of mesenchymal cells, isolated groups of adipocytes, blood vessels and neural structures within this layer.
− Week 27: In all samples, the deep fascia was composed of a huge cellularity without clear organization in layers (Figure 2B, Figure 3B, Figure 4B and Figure 6B). No sublayers were recognized, and in some cases, small aggregates of adipose tissue were found within these structures. The mean thickness was 319.8 ± 51.83 µm for all fascial structures, except for the extensor retinaculum of the upper limb, 169.7 ± 10.30 µm (Figure 7B).
− Week 29: In all samples, the deep fascia started to acquire an organization showing dense connective tissue (Figure 2C, Figure 3C, Figure 4C, Figure 5C and Figure 6C). Some of the sketches of the first layers began to show. The same histological features as those seen at 27 weeks were found, as described in samples from previous weeks. The mean thickness was 229.8 ± 87.79 µm for all fascial structures.
− Week 36: All samples started to show a well-differentiated deep fascia-like structure of 2–3 layers of dense regular connective tissue with areolar tissue in between (Figure 2D, Figure 3D, Figure 4D and Figure 6D). At this age, organization was seen in sublayers but the fibers were organized in one direction. The mean thickness was 239.3 ± 59.15 µm for all fascial structures.
− Week 38: As was seen at 36 weeks, the deep fascia was significantly clear, with a well-defined organization, with 2–3 layers of dense regular connective tissue with areolar tissue in between (Figure 2E, Figure 3E, Figure 4E, Figure 5C and Figure 6E). All samples showed a structure organized in different layers with different directions (transversal and longitudinal). The middle layer was the most prominent and consisted of compact bundles of collagen fibers, while the outermost layers showed a woven-like and thin pattern. This was evident in the deep fasciae of the anterior compartment of the thigh and the leg, but also in the iliotibial tract and in the extensor retinaculum of the ankle, while in the wrist the organization in layers has yet to be constituted. The mean thickness was 219.6 ± 86.1 µm for all fascial structures, except for the ex-tensor retinaculum of the upper limb, 334.6 ± 44.74 µm (Figure 7C).
− Week 40: The deep fascia shows a clear stratification. All samples showed a well-differentiated deep fascia-like structure of 2–3 layers of dense regular connective tissue with areolar connective tissue in between (Figure 2F, Figure 3F, Figure 4F, Figure 5D and Figure 6F). The connective tissue of the thigh region showed the most organized pattern, with the middle layer consisting of several non-aligned dense connective tissue bundles, all in the same direction. The mean thickness was 176.8 ± 58.95 µm for all fascial structures, except for the extensor retinaculum of the upper limb 545.4 ± 22.33 µm (Figure 7D).

#### 3.2.1. Hyaluronan and Elastic Fibers 

**Figure 9:**
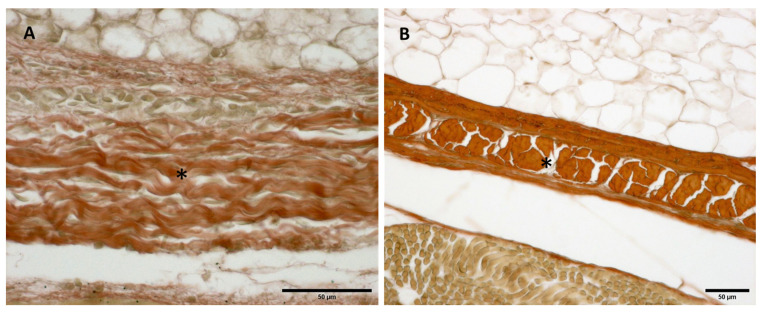
**Histological images (Weigert Van Gieson staining): Lateral deep fascia of the thigh:** (**A**) 24 weeks; (**B**) 38 weeks. (*): deep fascia. Elastic fibers, by Weigert Van Gieson staining, were present in a negligible percentage, less than 1% at all ages.

Alcian Blue staining and immunochemistry to detect HABP highlighted a huge amount of HA both in the deep fascia and fascial reinforcements in all the specimens, from 24 to 40 weeks of gestation (Figure 8). Major differences in distribution and organization were observed between samples from between weeks and gestation age at the extremes between them. The first part of the gestation is characterized, starting from the 24th week (Figure 8A,C), by less dense and irregular connective tissue with HA distributed in a disorganized manner, whilst the second part of the gestation, from the 36th week (Figure 8B,D), presented with a denser and compact connective tissue with HA distributed in an organized structure and which tends to diminish in quantity. In the first part of the gestation, HA was found randomly distributed in the deep fascia and in the fascial reinforcements, while in the second part of the gestation the HA was found to be distributed longitudinally within the whole layers of the connective tissue structure studied. Elastic fibers, by Weigert Van Gieson staining, were present in a negligible percentage, less than 1% (Figure 9).

#### 3.2.2. Collagen I and III

**Figure 10:**
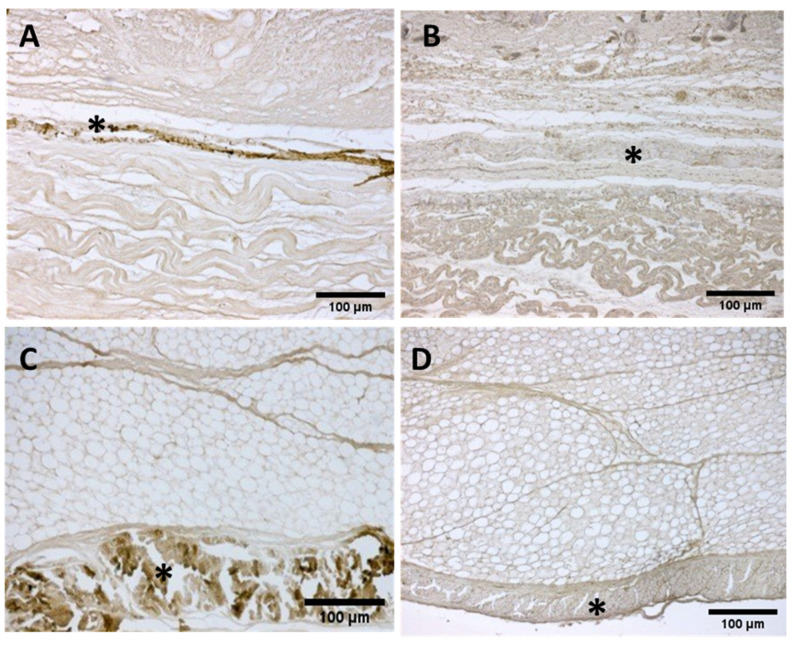
**Immunostaining to detect Collagens I and III:** Anterior deep fascia of the thigh: Collagen I (**A**) 29 weeks and (**C**) 38 weeks; Collagen III (**B**) 29 weeks and (**D**) 38 weeks. (*) deep fascia. The first part of gestation is distinguished, starting from the 29th week, by an irregular connective tissue with Col I and Col III beginning to delineate the layers (**A**,**B**), whilst the second part of the gestation, 38th week (**C**,**D**), presented with a denser connective tissue that was better organized, with Col I and Col III, which tend to increase in quantity and constitute the different fascial layers.

Immunochemistry to detect Collagen types I (Col I) and III (Col III) showed different amounts both in the deep fascia and fascial reinforcements in all the specimens, from 24 to 40 weeks of gestation (Figure 10). The most obvious differences in distribution and organization were noted between samples between weeks and gestation age at the extremes. The first part of gestation is distinguished, starting from the 29th week, by less and dense irregular connective tissue with Col I and Col III beginning to delineate the fascial directions and layers (Figure 10A,B), whilst the second part of the gestation, from the 36th week, presented with a denser connective tissue that was better organized, with Col I and Col III, which tend to increase in quantity and constitute the different fascial layers (Figure 10C,D). 

## 4. Discussion

To date, this is the first work examining the development of the fascial reinforcements, such as the iliotibial band and retinacula, in fetuses. The morphological features of the retinacula in the wrist and in the ankle studied showed similar features with the deep fascia, which tended to decrease in thickness, which was most evident in the iliotibial tract and extensor retinaculum of the ankle (Table 1). Moreover, in all-full thickness at 36th week of development, a precise organization of the deep fascia was seen, similar to those described in adults [18,19], while the samples collected around the joints did not present the thickening typical of the fascial reinforcements/retinacula, and only in those of the lower limb started to appear the organization in sublayers. 

As has been reported by other studies examining the deep fasciae of the forearm, thigh and lower back during fetal development [12,13,14,15,16], also in the retinacula in weeks 24–29, an irregular connective tissue runs parallel to the skin below the subcutaneous adipose tissue, with an organization in layers that appears at around 29–36 weeks in the ankle and 38 weeks in the wrist. The extensor retinacula of the wrist and ankle showed different developmental behaviors; the former had a huge thickness at the 40th week (735.3 ± 44.26 µm), much higher than the second (185.3 ± 94 µm). In light of these findings, it could be affirmed that movement determines the clear organization of these fascial reinforcements. Indeed, in the case of the hand, some authors reported much less movement and mechanical stimulations of the fetus with respect to the feet, and consequently probably the wrist retinacula develop late. Only after birth does the child start to use the hand in a complex manner; it is the maturation of fine motor skills, in the following months, that leads to the acquisition of voluntary grasping and the organization of the fascial reinforcements [20]. In addition, the smaller thickness of the extensor retinaculm of the ankle could have been determined by a different development of the motor skills of the ankle in the context of the fetal/general movements. Fetal joint movements start at seven weeks of gestation and isolated limb movements are identified until ten weeks [21]. Katz et al. [22] recorded in detail “a normal range of fetal knee movements until 32 weeks”. Shaw et al. [23] demonstrated that “the muscle contractions as well as joint movements are necessary for the development of the muscle-associated tendon and ligament. The joints of the fetuses at 25–33 weeks, are very active in utero”. Muscle contractions during this period might facilitate the different configurations of the extensor ankle retinaculum, the iliotibial tract, anterior thigh fascia, anterior crural fascia and posterior antebrachial fascia, in accordance with the motor tasks used by the fetus during gestation [20]. This evidence could explain the different thicknesses between the extensor retinacula of the ankle and of the wrist. The fetal movement of the lower limb is more predominant than the upper limb, explaining the greater thickness of the deep fascia in the upper limb than the lower limb; the latter starts to “run” particular patterns of movement and delineate their structure [21]. A greater thickness does not mean it is stronger, but more mesenchyme means more HA, without defined fiber bundles, and therefore looks like a white tablet. The acquisition of the ability to use the upper limbs and in particular the hands is a motor task that is acquired after birth [20]. 

Moreover, HA is abundant and randomly distributed in the first part of gestation (24th–29th), while in the second part of gestation (36th–40th) it was found to be distributed longitudinally within the whole layers of the fascial structures studied. These findings are consistent with those of other studies that reported the crucial role of HA in the fascial layers, permitting their gliding during the movement and the feature to reduce it with the age [24,25]. 

Finally, an analysis of our results showed that the quantity of elastic fibers, during gestation, is negligible. As reported by Pirri et al. [19], “Elastic component, inside loose connective tissue, allows the fibrous layers to move with respect to the adjacent one and to return to initial”, supporting the hypothesis that the movement, the load and the gravity stimulate their development in the deep fascia mixing their elastic capacity and of force transmission (Appendix A).

An analysis of our study results showed that there was a different trend in the topographical regions of the retinacula than the posterior forearm fascia (antebrachial fascia), the anterior thigh fascia (fascia lata), anterior leg fascia (crural fascia) and the iliotibial tract. The deep fascia was clearly identified already at 24 weeks in the lateral region of thigh, confirming the closed relationship between mechanical stress and the development of the fasciae. Moreover, the thickness of the iliotibial tract is similar to the anterior part of the fascia lata (118.3 ± 15.3 µm vs. 160.40 ± 6.1 µm), whilst in adults the thicknesses tend to be higher laterally [18]; all this could be determined by the absence of gravity and load, which play the important role in determining the lines of myofascial force transmission in these compartments, compatibly to the role of the attachment and myofascial expansion of the thigh muscles [18]. 

The disposition of collagen fibers in the iliotibial tract was initially transversally and longitudinally oriented, to then become predominant in the longitudinal direction (Figure 4). This evidence could explain, from a translational point of view, the role of fetal movement in the fetal fascial organization. The individual differences might depend on the frequency and range of joint movements in utero [15,20]. 

Despite the study needing to be deepened, in light of these findings the possible role of the gestational movement in the development of the retinacula, deep fascia of anterior thigh, iliotibial tract, posterior part of the antebrachial fascia and the anterior crural fascia is evident. The retinacula can sense bone movement and muscular contraction due to their connections to specific muscular contraction and bony areas [26,27,28,29,30,31]. The continuous mechanical load in newborns stimulates the deposition of new collagen fibers along specific directions, resulting in the formation of retinacula around joints and in the selection of particular directions of movement [13,20]. This also suggests that the movement of the fetus stimulates, in a different manner, the retinacula of the ankle, the wrist, iliotibial tract, anterior thigh fascia, anterior crural fascia and posterior antebrachial fascia. 

To the best of our knowledge, the present study is the first report showing how the features of the retinacula of wrist and ankle, iliotibial tract and anterior thigh fascia, antebrachial and crural fascia change over the prenatal period. An important change is likely to occur in the original fascial configuration, such as the organization of the deep fascia layers, due to the fetal movement in utero. 

### Limitations of the Study

The small number of fetal cadaveric body donors included in this study cohort and the qualitative aspect of the assessments mean that it is not possible to statistically analyze the prevalence of anatomical findings. Additional studies and data about this topic would help to establish a stronger correlation between the fetal and adult retinacula and would broaden our understanding of the myofascial system. A better understanding of the histological features of these fascial components in development can better explain the involvement of these in pediatric and musculoskeletal diseases, both in structural and sometimes in functional alterations, creating new types of treatment in the rehabilitation field and beyond.

## 5. Conclusions

To conclude, our study results confirm that it is possible to visualize the fasciae in the fetus and also the retinacula. This study added to the knowledge about the fetal development of the retinacula. Our results may contribute to a better understanding of the development of deep fasciae and their fibrous reinforcements, improving the knowledge about the difference between fascial reinforcements and deep/muscular fascia. Accordingly, this difference may explain the role of fetal movement in deep fasciae development. Considering the potential applications of this knowledge, it is important to assess gestational movements and movement ability in the first months of life, highlighting any dysfunctions at the fascial level. In the field of the rehabilitation and pediatrics, fasciae can be an important target of therapy and rehabilitation in childhood disabilities. The fasciae can be imagined, initially, as “white tablets” composed of few elastic fibers, abundant collagens and HA, on which the various forces, movements, loads and gravity, “write their history”.

## Figures and Tables

**Table 1:** Fascial reinforcement and deep fascia: the measurements are expressed in µm.

Weeks	Upper Limb (Extensor Retinaculum)	Upper Limb (Antebrachial Fascia)	Lower Limb (Extensor Retinaculum)	Lower Limb (Crural Fascia)	Lower Limb (Anterior Thigh Fascia):	Lower Limb (Iliotibial Tract)
24	112.40 ± 6.1	652.73 ± 187.6	514.1 ± 140	486.3 ± 116.1	735.3 ± 44.26	269.4 ± 31.80
27	169.7 ± 10.30	328.38 ± 59.38	340 ± 40.41	365.5 ± 79.1	334.6 ± 44.74	230.6 ± 10.3
29	-	310.1 ± 41.22	162 ± 23	311 ± 18	285.4 ± 16.4	280.59 ± 5.84
36	-	292.54 ± 118.4	195 ± 21.04	305.1 ± 30	234.3 ± 12	368.2 ± 51.64
38	334.6 ± 44.74	364.83 ± 93.25	190 ± 23.02	225.7 ± 45	169.7 ± 10.30	147.8 ± 33.53
40	545.4 ± 22.33	147.11 ± 49.04	185.3 ± 94	273 ± 18.44	160.40 ± 6.1	140.3 ± 13.4

**Figure A1:**
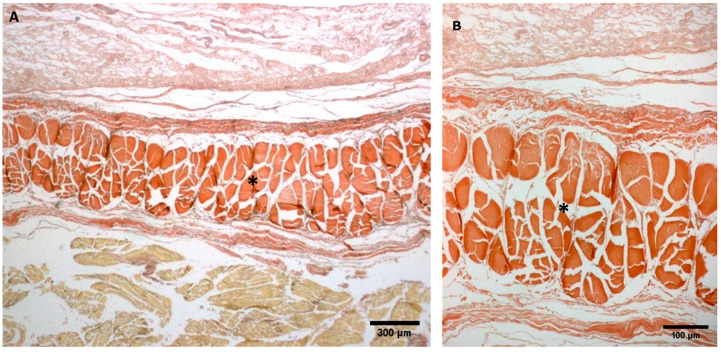
(**A**,**B**): Histological images (Weigert Van Gieson staining): (*) Lateral deep fascia of the thigh in adult.

## Data Availability

The data presented in this study are available on request from the corresponding author.

## Abbreviations

Hyaluronic Acid-Binding Protein: HABP; Collagen I: Col I; Collagen III: Col III.
